# The Association Between Long-Term Exposure to Particulate Matter and Incidence of Hypertension Among Chinese Elderly: A Retrospective Cohort Study

**DOI:** 10.3389/fcvm.2021.784800

**Published:** 2022-01-11

**Authors:** Zhou Wensu, Chen Wen, Zhou Fenfen, Wang Wenjuan, Ling Li

**Affiliations:** Department of Medical Statistics, School of Public Health, Sun Yat-sen University, Guangzhou, China

**Keywords:** China, hypertension, elderly, particulate matter, cohort study

## Abstract

**Background and Objectives:** Studies that investigate the links between particulate matter ≤2. 5 μm (PM_2.5_) and hypertension among the elderly population, especially those including aged over 80 years, are limited. Therefore, we aimed to examine the association between PM_2.5_ exposure and the risk of hypertension incidence among Chinese elderly.

**Methods:** This prospective cohort study used 2008, 2011, 2014, and 2018 wave data from a public database, the Chinese Longitudinal Healthy Longevity Survey, a national survey investigating the health of those aged over 65 years in China. We enrolled cohort participants who were free of hypertension at baseline (2008) from 706 counties (districts) and followed up in the 2011, 2014, and 2018 survey waves. The annual PM_2.5_ concentration of 706 counties (districts) units was derived from the Atmospheric Composition Analysis Group database as the exposure variable, and exposure to PM_2.5_ was defined as 1-year average of PM_2.5_ concentration before hypertension event occurrence or last interview (only for censoring). A Cox proportional hazards model with penalized spline was used to examine the non-linear association between PM_2.5_ concentration and hypertension risk. A random-effects Cox proportional hazards model was built to explore the relationship between each 1 μg/m^3^, 10 μg/m^3^ and quartile increment in PM_2.5_ concentration and hypertension incidence after adjusting for confounding variables. The modification effects of the different characteristics of the respondents were also explored.

**Results:** A total of 7,432 participants aged 65–116 years were enrolled at baseline. The median of PM_2.5_ exposure concentration of all the participants was 52.7 (inter-quartile range, IQR = 29.1) μg/m^3^. Overall, the non-linear association between PM_2.5_ and hypertension incidence risk indicated that there was no safe threshold for PM_2.5_ exposure. The higher PM_2.5_ exposure, the greater risk for hypertension incidence. Each 1 μg/m^3^ [adjusted hazard ratio (AHR): 1.01; 95% CI: 1.01–1.02] and 10 μg/m^3^ (AHR: 1.12; 95% CI: 1.09–1.16) increments in PM_2.5_, were associated with the incidence of hypertension after adjusting for potential confounding variables. Compared to first quartile (Q1) exposure, the adjusted HRs of hypertension incidence for the Q2, Q3 and Q4 exposure of PM_2.5_ were 1.31 (95% CI: 1.13–1.51), 1.35 (95% CI: 1.15–1.60), and 1.83 (95% CI: 1.53–2.17), respectively. The effects appear to be stronger among those without a pension, living in a rural setting, and located in central/western regions.

**Conclusion:** We found no safe threshold for PM_2.5_ exposure related to hypertension risk, and more rigorous approaches for PM_2.5_ control were needed. The elderly without a pension, living in rural and setting in the central/western regions may be more vulnerable to the effects of PM_2.5_ exposure.

## Introduction

Hypertension is the most prevalent chronic disease among the elderly (1) and has led to adverse cardiovascular diseases (CVDs), such as hypertensive heart disease and stroke, and even death (2). Worldwide, as the elderly population continues to grow, the disease burden attributed to hypertension and its complications are increasing (3, 4) so it is imperative to identify risk factors for hypertension and promote prevention among the elderly.

In addition to well-established associations with lifestyle and heredity, it is thought that environmental pollutants also contribute to the occurrence of hypertension (5, 6). In particular, the inhalation of air containing fine particulate matter (PM), especially sizes ≤2.5 um (PM_2.5_) has been reported as a probable antecedent driver of the increase in blood pressure and incidence of hypertension (7–12). Overall, although the association between PM_2.5_ exposure and hypertension has been reported in several studies, gaps in knowledge remain. For example, elderly people may be more susceptible to PM_2.5_, due to higher rates of CVDs and given the decline in organ function associated with age (13, 14). However, relevant studies rarely focus on the elderly, especially those aged over 80 years (15–19).

Furthermore, inconsistent results have been reported in previous studies. For instance, based on a cross-sectional study involving 27,752 Taipei City residents (mean age 74.8 years), Chen et al. found that PM_2.5_ exposure was not significant associated with diastolic blood pressure (DBP) and none of the air pollutants were associated with changes in systolic blood pressure (SBP) (16). In addition, cross-sectional study designs and small-scale sample sizes limit the generalizability of the results to the elderly population (17, 19–21).

Lastly, many studies have been conducted in regions and countries, such as Taiwan and the USA (16, 21), with good air quality defined by PM_2.5_ concentrations lower than the World Health Organization recommendations (10 μg/m^3^). It has been suggested that the conclusions drawn from studies conducted in other regions might not be suitable for generalization to China (11, 22). China has some of the heaviest air pollution in the world with an average annual population-weighted PM_2.5_ exposure of 52.7 μg/m3 in 2017 (23). More importantly, PM_2.5_ has been proven to be a modifiable factor that contributes to cardiovascular morbidity and mortality (24). Thus, considering the research gaps and public health significance, it is necessary to study and verify the effects of PM_2.5_ on hypertension among the elderly to provide more effective interventions for the elderly and medical resource allocation.

In the present prospective cohort study based on the national representative Chinese Longitudinal Healthy Longevity Survey (CLHLS), we aimed to examine the association between PM_2.5_ exposure and the risk of hypertension incidence among Chinese elderly. Secondarily, we aimed to further explore the modifying effects of PM_2.5_ exposure on hypertension incidence and identify vulnerable sub-populations.

## Materials and Methods

### Study Population

This was a prospective cohort study that selected data from the 2008, 2011, 2014, and 2018 waves of the CLHLS. The CLHLS is a nationwide survey that covers 23 of 31 provinces, municipalities, and autonomous regions in mainland China (the remaining provinces are not surveyed because of their low level of population density), which includes 85% of the total population of the country (25). The CLHLS was established in 1998 with enrollment of the elderly population (aged 65 years and older) and traced them in 1998, 2000, 2002, 2005, 2008, 2011, and 2018. In the 2008 wave, the CLHLS added data collection on individuals' community-level information, including economic development, the natural environment, and environmental pollution. The CLHLS study was approved by the Institutional Review Board of the Duke University Health System and all participants sign an informed consent form. A detailed introduction to the CLHLS has been published previously (26).

**Figure 1:**
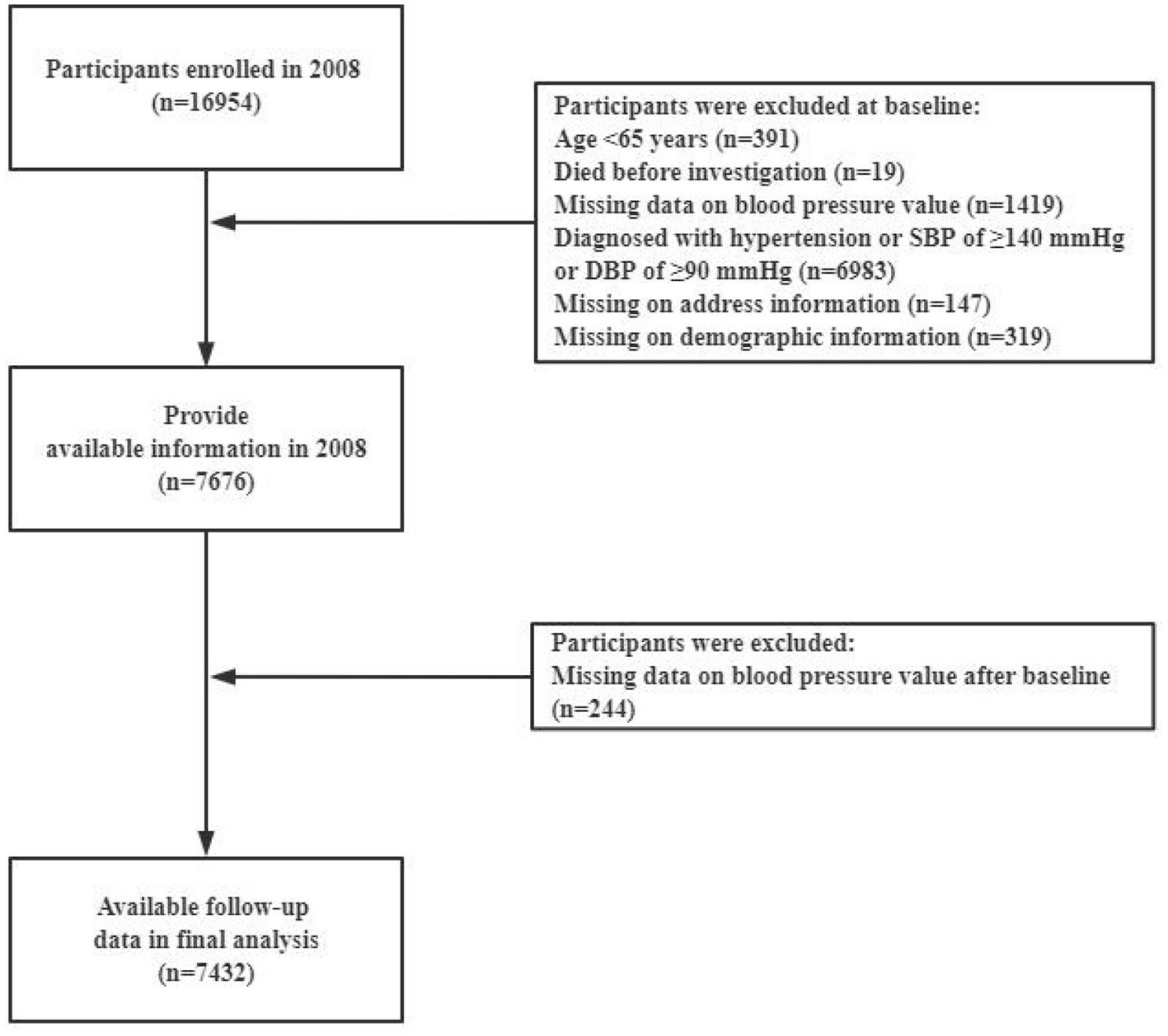
The flow chart of study population selection.

The cohort for the present study was derived from 10 years of follow-up (2008–2018) from the 2008 baseline when the availability of county (district) unit level address information became available. The cohort comprised 7,432 participants from 706 county (district) units aged 65–116 years. The participants were all free of hypertension (normal blood pressure at the time of the survey and self-reported as not being diagnosed with hypertension by a physician) and had complete demographic characteristic information at baseline in 2008. They were interviewed in 2011, 2014, and 2018. A flow chart of the study population selection is shown in Figure 1. The follow-up duration was reported as person-years calculated by using days divided by 365 from the date of study enrollment to the date of last interview, death, or hypertension incidence (whichever came first). The outcome of the event was considered as the occurrence of hypertension, while other conditions such as death, lost follow-up, and not been identified as hypertension were censored data.

### Assessment of Hypertension

As part of the CLHLS study, blood pressure values for each participant were collected by trained investigators using a mercurial sphygmomanometer (upper arm type; Yuyue, Jiangsu, China) at baseline and at every follow-up interview. Their blood pressure was measured two times after a five or more min rest period. Specifically, for bedbound participants, blood pressure measurements were obtained in a recumbent position. SBP and DBP values were calculated as the average of two repeated measurements. According to the Chinese Guidelines for Prevention and Treatment of Hypertension (27), we defined hypertension by an SBP ≥ 140 mmHg, DBP ≥ 90 mmHg, or normal blood pressure with a self-reported hypertension diagnoses previously made by a grade II or III hospital. The definition of hypertension was supported by prior study using same database (28).

### Assessment of PM_2.5_ Exposure

Due to the privacy protection, the information of participant's home address was deleted in the CLHLS. Thus, we identified the 706 residence name [i.e., county (district) from community environment questionnaire in this study], detailed strategy was published in a prior study (29). The annual average residential PM_2.5_ concentration from 706 residential county (district) units of the 7,432 participants was collected through an open database built by the Atmospheric Composition Analysis Group from the University of Washington (https://sites.wustl.edu/acag/) (30). The database collects ground-level PM2.5 measurements were obtained from http://beijingair.sinaapp.com/ over mainland China. These data are captured by individuals from instantaneous data records on the website of the Chinese EPA. The PM_2.5_ was evaluated from satellite observations with 0.01 spatial resolution (1.1 × 1.1 km). The resultant PM_2.5_ estimates were highly convergent (*R*^2^ = 0.81) with out-of-sample cross-validated PM_2.5_ concentrations from monitors (31). This database has been widely used in previous studies (22, 32).

Referring to prior studies (33, 34), the PM_2.5_ exposure was defined as 1-year average before the hypertension event occurrence or the last interview (only for censored data). This exposure time was selected because it has the strongest hazard risk for hypertension (Supplementary Table 1). In our study, only 116 (1.5%) of participants moved to another address (county/distrust level), which was similar to prior study (22). Thus, we hypothesized that the PM_2.5_ exposure of the population was stable, we also performed sensitivity analysis through excluding participants who changed address (Supplementary Table 3).

### Potential Confounding Variables

According to previous studies, several demographic, lifestyle, and health status variables at baseline were considered potential confounding factors and adjusted for, including sex (male or female), residence (rural or urban areas), age (65–80 or >80 years), regions (eastern China or central/western China), living arrangement, pension (yes or no), educational attainment (0 or >0 years), marital status (separated/divorced/never married, widowed, or married), body mass index (BMI) (<18.5, 18.5–23.9, or >23.9), smoking at the present (yes or no), drinking at the present (yes or no), exercising at the present (yes or no), history of diabetes (yes or no), heart disease (yes or no) and function disability. Among them, function disability was measured by Activities of daily living (ADL) scale (35). The scale has six items including the fundamental skills: bathing, dressing, eating, toileting, continence, and transferring. If a respondent is able to perform an activity, he/she gets score 1, and if he/she is limited to do and unable to do so, will get score 2 and 3, respectively. The more scores of the individual, indicates poor ADL ability. According to the cutoff value (six points), the function disability was set as binary variable in the analysis (yes or no).

The per capita gross domestic product (GDP) (≥median or < median) and proportion of secondary industry (≥median or < median) in the city level at baseline were also considered as confounding variables in the analysis.

### Statistics Analysis

A descriptive analysis was conducted for all the variables. Continuous variables are expressed as mean (±SD) or median (interquartile range). Categorical variables are reported as numbers and percentages. We conducted a statistical map to describe the spatial distribution of PM_2.5_ concentration in 706 (counties or districts) unit-level residences at baseline. To identify the potential linear or non-linear relationship between PM_2.5_ exposure and hazard ratios (HRs) of hypertension incidence, we built a Cox proportional hazards model with penalized splines and different degrees of freedom based on the minimum value of Akakai information criteria (AIC) to visualize the exposure-response relationship between PM_2.5_ and hypertension. Because the multiple-level structure of CLHLS showed the clustering of participants at the study level (counties or districts) units, we used a random-effects Cox proportional hazards model for the clustering level to explore the relationship between PM_2.5_ exposure and hypertension incidence among the elderly. We classified PM_2.5_ concentration in increments of 1 and 10 μg/m^3^. The estimation of the risk of hypertension incidence based on 1 and 10 μg/m^3^ increments in PM_2.5_ concentration were conducted using the random-effects Cox proportional hazards model with PM_2.5_ exposure as a continuous variable. According to quartiles, PM_2.5_ was categorized into four groups (Q1: ≤ P_25_, Q2: (P_25_-P_50_], Q3: (P_50_-P_75_], and Q4: >P_75_), and the first group (Q1) was coded as a reference to examine the association between exposure and hypertension incidence. The crude model only included the PM_2.5_ variable. Model 1 further added age, sex, educational attainment, pension, living arrangement, marital status, regions, residence, smoking at the present, BMI, drinking at present, exercising at the present, self-reported diabetes, function disability, GDP per capita, the proportion of secondary industry and heart disease. Analysis was also performed to examine the linear trend between the PM_2.5_ quartile and hypertension incidence. The HRs and 95% confidence intervals (CIs) were calculated to evaluate the effects of PM_2.5_ exposure on hypertension after adjusting for potential variables.

We also conducted subgroup analysis to evaluate whether the effect of PM_2.5_ exposure on hypertension incidence differed by sex, age, educational attainment, living arrangement, residence, pension, marital status, regions, smoking at the present, drinking alcohol at the present, exercising at the present, BMI, self-reported diabetes, function disability, GDP per capita, the proportion of secondary industry and heart disease after adjusting for related covariates. Referring to a prior study (36), a 2-sample test for assessing statistically significant differences in the estimated HR within each subgroup was performed using the point estimate and standard error (SE) in this study (see Formula ①).


(1) $$\begin{array}{r} Z=\frac{Q_{1}-Q_{2}}{\sqrt{{\left(SE_{1}\right)}^{2}+{\left(SE_{2}\right)}^{2}}} \end{array}$$

In the equation, Q_1_ and Q_2_ are the estimated hazard ratios for each stratum, respectively. SE_1_ and SE_2_ are the standard errors for each stratum, respectively.

The map of China was derived from National Geomatics Center of China (http://www.ngcc.cn/ngcc/), the statistics map was generated by ArcGIS Geospatial Analyst module v10.6 (ESRI, Redlands, CA, USA). All analyses were performed using R statistical software (R 4.0.5, R Foundation for Statistical Computing, Vienna, Austria). A two-sided *P*-value < 0.05 was used to assess statistical significance.

## Results

At baseline, 7,432 participants were enrolled in the study. During the 10-year follow-up, the total number of person-years was 24,222 and the incidence of hypertension was 8.5 per 100 person-years. The demographic characteristics, lifestyles, and health status of the participants from the CLHLS at baseline are presented in Table 1. The mean age of the participants was 87.7 (±11.5) years. Of the elderly, 56.4% were female and 62.6% were uneducated. Over half of them had no pension. Of the participants, 80.8% lived in rural areas and 83.7% lived with family members. More participants came from western/central China than from eastern China. A total of 17.6 and 18.4% of them had smoking and drinking habits, respectively. A total of 27.4% of the participants reported that they had a habit of exercising at the present. Only 1.7% and 5.8% of the patients reported having diabetes and heart disease, respectively. A total of 52.7% of the participants had a BMI between 18.5 and 23.9. Most of them without function disability. In our study, the range of PM_2.5_ exposure was from 3.8 to 134.8 μg/m^3^. The median 1-year averages were PM_2.5_ = 52.7, P_25_ = 41.1 μg/m^3^, and P_75_ = 70.1 μg/m^3^.

**Table 1:** The demographic characteristics of the participants from the CLHLS at baseline (*n* = 7,432). Data are number (%) of participants except PM _2.5_ concentration [median (IQR)].

**Characteristic of participants in the study**		**Entire cohort**	**PM_2.5_**			
			**Q1**	**Q2**	**Q3**	**Q4**
Gender						
	Female	4,193 (56.4)	992 (53.4)	1,047 (56.2)	1,079 (58.2)	1,075 (57.9)
	Male	3,239 (43.6)	866 (46.6)	816 (43.8)	774 (41.8)	783 (42.1)
Age (year)						
	65–80	2,141 (28.8)	644 (34.7)	548 (29.4)	500 (27.0)	449 (24.2)
	>80	5,291 (71.2)	1,214 (65.3)	1,315 (70.6)	1,353 (73.0)	1,409 (75.8)
Education level (year)						
	0	4,654 (62.6)	1,120 (60.3)	1,096 (58.8)	1,157 (62.4)	1,281 (68.9)
	>0	2,778 (37.4)	738 (39.7)	767 (41.2)	696 (37.6)	577 (31.1)
Pension						
	No	6,162 (82.9)	1,573 (84.7)	1,536 (82.4)	1,486 (80.2)	1,567 (84.3)
	Yes	1,270 (17.1)	285 (15.3)	327 (17.6)	367 (19.8)	291 (15.7)
Residence						
	Rural	6,004 (80.8)	1,537 (82.7)	1,589 (85.3)	1,436 (77.5)	1,442 (77.6)
	Urban	1,428 (19.2)	321 (17.3)	274 (14.7)	417 (22.5)	416 (22.4)
Living arrangement						
	Nursing institution/alone	1,210 (16.3)	322 (17.3)	354 (19.0)	260 (14.0)	274 (14.7)
	Living in home with family member (s)	6,222 (83.7)	1,536 (82.7)	1,509 (81.0)	1,593 (86.0)	1,584 (85.3)
Regions						
	Western/central China	4,477 (60.2)	1,064 (57.3)	1,188 (63.8)	856 (46.2)	1,369 (73.7)
	Eastern China	2,955 (39.8)	794 (42.7)	675 (36.2)	997 (53.8)	489 (26.3)
Current marital status						
	Widowed/separated/ divorced/never married	5,112 (66.8)	1,179 (63.5)	1,254 (67.3)	1,327 (71.6)	1,352 (72.8)
	Married	2,320 (31.2)	679 (36.5)	609 (32.7)	526 (28.4)	506 (27.2)
Smoking at the present						
	No	6,124 (82.4)	1,557 (83.8)	1,543 (82.8)	1,497 (80.8)	1,527 (82.2)
	Yes	1,308 (17.6)	301 (16.2)	320 (17.2)	356 (19.2)	331 (17.8)
Drink alcohol at the present						
	No	6,063 (81.6)	1,548 (83.3)	1,504 (80.7)	1,506 (81.3)	1,505 (81.0)
	Yes	1,369 (18.4)	310 (16.7)	359 (19.3)	347 (18.7)	353 (19.0)
Exercising at the present						
	No	5,397 (72.6)	1,330 (71.6)	1,328 (71.3)	1,380 (74.5)	1,359 (73.1)
	Yes	2,035 (27.4)	528 (28.4)	535 (28.7)	473 (25.5)	499 (26.9)
Diabetes						
	No	7,304 (98.3)	1,838 (98.9)	1,829 (98.2)	1,811 (97.7)	1,826 (98.3)
	Yes	128 (1.7)	20.0 (1.1)	34.0 (1.8)	42.0 (2.3)	32.0 (1.7)
Heart disease						
	No	7,002 (94.2)	1,746 (94.0)	1,793 (96.2)	1,737 (93.7)	1,726 (92.9)
	Yes	430 (5.8)	112 (6.0)	70.0 (3.8)	116 (6.3)	132 (7.1)
BMI index						
	>23.9	773 (10.4)	164 (8.8)	158 (8.5)	197 (10.6)	254 (13.7)
	18.5–23.9	3,915 (52.7)	943 (50.8)	923 (49.5)	1,043 (56.3)	1,006 (54.1)
	<18.5	2,744 (36.9)	751 (40.4)	782 (42.0)	613 (33.1)	598 (32.2)
Function disability						
	Yes	1,559 (21.0)	315 (16.9)	441 (23.8)	470 (25.3)	315 (16.9)
	No	5,873 (79.0)	1,548 (83.1)	1,412 (76.2)	1,388 (74.7)	1,548 (83.1)
GDP per capita						
	≥P_50_	3,727 (50.1)	845 (45.5)	895 (48.0)	1,118 (60.3)	869 (46.8)
	< P_50_	3,705 (49.9)	1,013 (54.5)	968 (52.0)	735 (39.7)	989 (53.2)
The proportion of secondary industry						
	≥P_50_	3,910 (52.6)	941 (50.6)	835 (44.8)	1,053 (56.8)	1,081 (58.2)
	< P_50_	3,522 (47.4)	917 (49.4)	1,028 (55.2)	800 (43.2)	777 (41.8)
	PM _2.5_ concentration	52.7 (29.1)	34.7(7.3)	46.2(5.8)	60.7(7.7)	80.4 (11.4)

**Figure 2:**
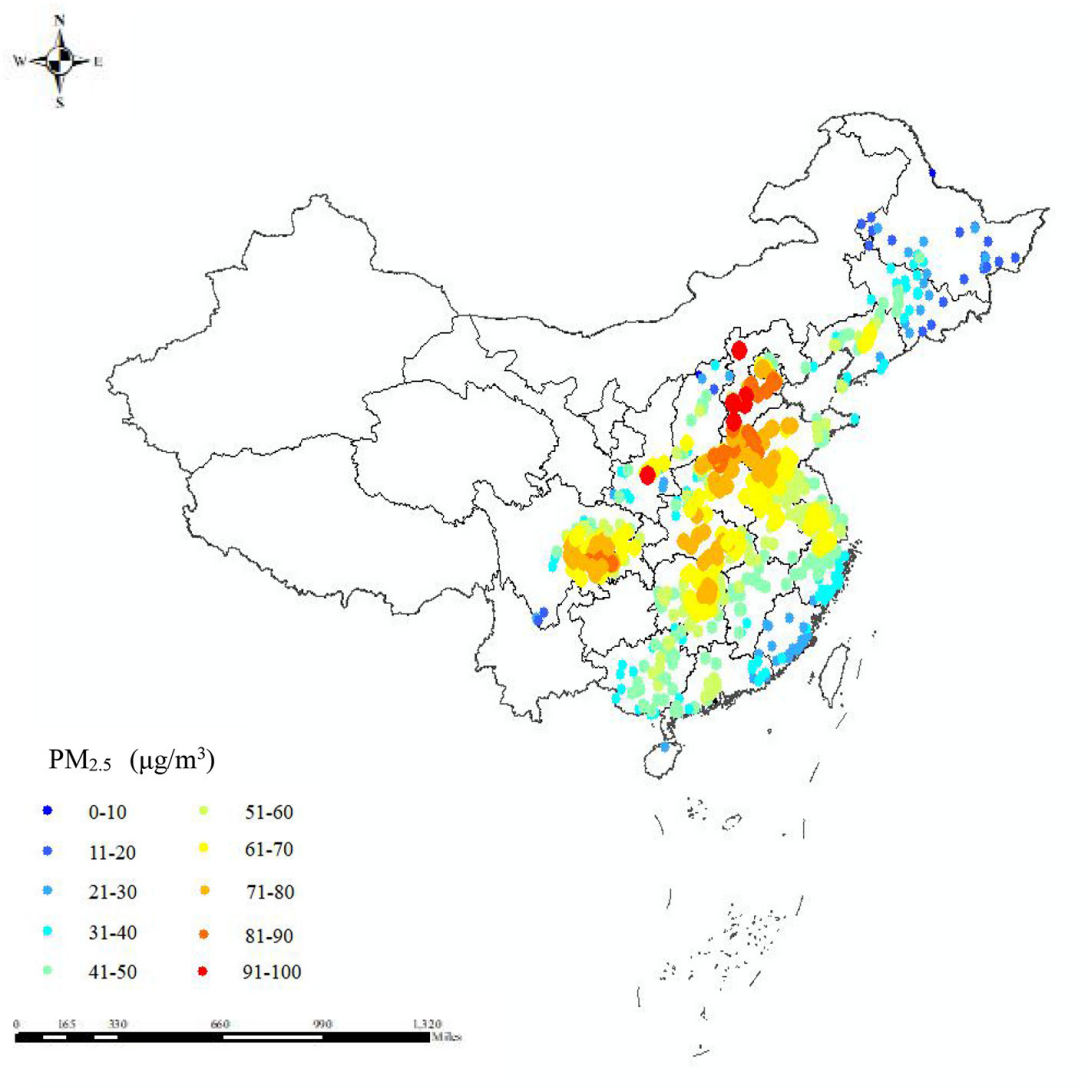
Statistic map presenting the residential PM_2.5_ exposure in 706 counties (districts) units of China at baseline for 7,432 elderly from the CLHLS.

The distribution of residential PM_2.5_ exposure of the 706 counties (districts) units at baseline is shown by a statistical map (Figure 2). The geographical distribution of PM_2.5_ concentration was higher in the north than the south, and higher inland than the coastal areas. In addition, the areas with high PM_2.5_ concentrations (>61 μg/m^3^) included the Sichuan Basin, Beijing-Tianjin-Hebei region, Shandong province, Hunan province, and Hubei province.

**Figure 3:**
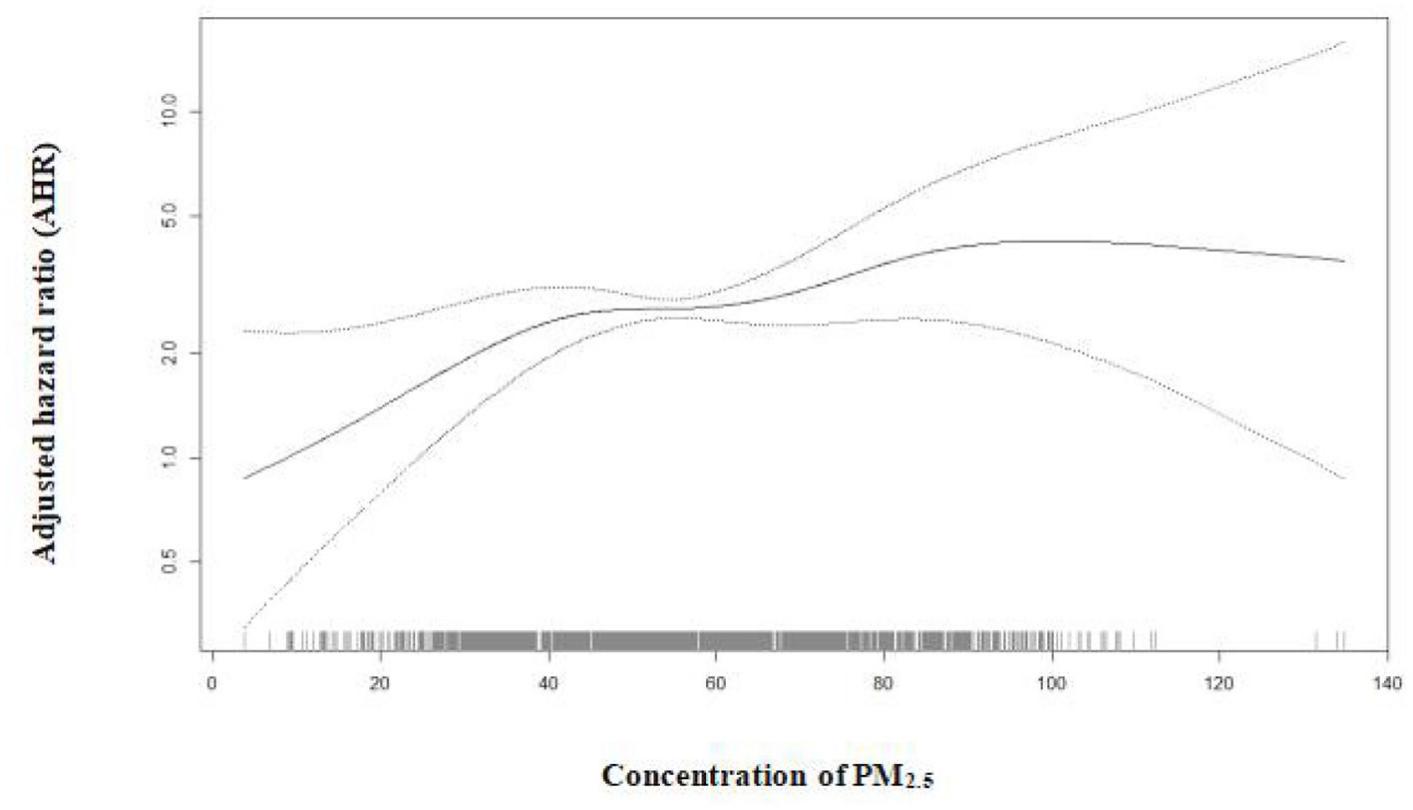
The nonlinear relationship between a 10 μg/m3 increase in PM_2.5_ exposure and hypertension based on Cox proportional hazards model with penalized splines of 4 degree of freedom (*P* for non-linear association was 0.008 and the non-linear relationship as reference = 3.80 μg/m3) adjusted for age, gender, education attainment, pension, living arrangement, marital status, regions, residence, smoking at the present, drinking at the present, exercising at the present, BMI index, self-reported diabetes, function disability, GDP per capita, the proportion of secondary industry and heart disease.

The Cox proportional hazards model with 4 degrees of freedom (the minimum AIC value) showed that there was a non-linear association between PM_2.5_ exposure and hypertension with 3.8 μg/m^3^ as the reference group since it was the lowest concentration of PM_2.5_ exposure (Figure 3). We observed strong evidence of linearity at concentrations below 50.1 μg/m^3^ and a stable increase in hazard risk between concentrations of 50.1 and 60.1 μg/m^3^. A steeper slope was found at 60.1 μg/m^3^ concentration and higher. Specifically, the hypertension risk was minor decreased over 107.6 μg/m^3^, but the effect was negligible because very little sample (accounting for 0.2% of total sample) over this value.

The incidence of hypertension in the first quartile, Q2, Q3, and Q4 were 9.3, 8.8, 7.3, and 8.5 per 100 person-years, respectively (Table 2). The results of the effects of PM_2.5_ exposure and hypertension incidence using a random-effects Cox proportional hazards model are presented in Table 3. Adjusting for potential confounding variables such as age, sex, educational attainment, and pension et al., we found that a 1 μg/m^3^ increment in PM_2.5_ concentration was associated with hypertension incidence [adjusted HR (AHR): 1.01, 95% CI: 1.01–1.02] and per 10 μg/m^3^ increment in PM_2.5_, the concentration was associated with hypertension incidence (AHR: 1.12; 95% CI: 1.09–1.16). Similar results were observed according to quartiles, where higher concentrations were strongly related with hypertension incidence (Q2 AHR: 1.31, 95% CI: 1.13–1.51; Q3 AHR: 1.35, 95% CI: 1.15–1.60; Q4 AHR: 1.83, 95% CI: 1.53–2.17). Trend analysis showed that there was a linear trend between the PM_2.5_ concentration quartile and hypertension incidence (*P* trend <0.001).

**Table 2:** The incidence rate for the effects of annual average residential PM_2.5_ exposure on hypertension among the elderly for 10-year cohort study.

**The Quartile of PM_**2.5**_ exposure**	**Person-year**	**Cases**	**Incidence rate (per 100 person years)**
Q1	7,118.2	661	9.3
Q2	6,048.9	533	8.8
Q3	5,397.1	394	7.3
Q4	5,657.9	478	8.5

**Table 3:** The association between long-term exposure to PM_2.5_ and hypertension incidence among the elderly.

**Models**	**PM**_**2.5**_				***P* for trend**	**PM**_**2.5**_	
	**Q1**	**Q2**	**Q3**	**Q4**		**Per 1-μg/m^**3**^ increment**	**Per 10-μ g/m^**3**^ increment**
Crude model	1	1.31 (1.13–1.51)	1.35 (1.15–1.60)	1.77 (1.49–2.09)	<0.001	1.01 (1.01–1.12)	1.12 (1.09–1.16)
Adjusted Model 1	1	1.31 (1.13–1.51)	1.35 (1.15–1.60)	1.83 (1.53–2.17)	<0.001	1.01 (1.01–1.12)	1.12 (1.09–1.16)

*Crude model: random-effects Cox proportional hazards model for the sampling sites and only included PM_2.5_ exposure*.

*Adjusted Model 1: random-effects Cox proportional hazards model for the sampling sites further adjusted for age, gender, education attainment, pension, living arrangement, marital status, regions, residence, smoking at the present, drinking at the present, exercising at the present, BMI index, self-reported diabetes, function disability, GDP per capita, the proportion of secondary industry and heart disease*.

In subgroup analyses, after adjusting for related confounding variables, the effect estimate for each 10 μg/m^3^ increment in PM_2.5_ concentration was significantly greater in individuals with without a pension, living in a rural setting, and residing in central/western China (*P*-value for modification effect <0.05 and significant HR value). The detailed modification effects are shown in Table 4.

**Table 4:** Subgroup analysis for hypertension incidence and its associated with each 10 μg/m^3^ increment in PM_2.5_ concentration.

**Characteristic**	**Groups**	**Person year**	**Cases**	**Incidence of hypertension (per 100 person year)**	**HR (95%CI)**	***P*-value for effect modification**
Age						
	65–80	9,663.53	1,014	10.49	1.26 (1.21–1.32)	(Reference)
	>80	14,558.56	1,052	7.23	0.98 (0.94–1.02)	0.999
Gender						
	Male	11,096.59	937	8.44	1.10 (1.05–1.44)	(Reference)
	Female	13,125.50	1,129	8.60	1.06 (1.02–1.10)	0.921
Education attainment						
	0	13,968.92	1,153	8.25	1.07 (1.03–1.11)	(Reference)
	>0	10,253.17	913	8.90	1.10 (1.05–1.15)	0.145
Living arrangement						
	Living in home with family member (s)	20,241.03	1,703	8.41	1.11 (1.07–1.15)	(Reference)
	Nursing institution/alone	3,981.06	363	9.12	1.04 (0.98–1.10)	0.974
Pension						
	Yes	4,330.60	340	7.85	1.00 (0.94–1.07)	(Reference)
	No	20,241.03	1,726	8.53	1.15 (1.11–1.19)	<0.001
Marital status						
	Married	9,423.61	918	9.74	1.15 (1.10–1.20)	(Reference)
	Widowed/separated/ divorced/never married	14,798.48	1,148	7.76	1.03 (0.99–1.07)	0.999
Regions						
	Eastern	9,560.80	819	8.57	1.02 (0.97–1.07)	(Reference)
	Central/Western	14,661.28	1,247	8.51	1.22 (1.16–1.27)	<0.001
Residence						
	Urban	4,428.99	268	6.05	1.01 (0.94–1.08)	(Reference)
	Rural	19,793.10	1,798	9.08	1.16 (1.12–1.20)	0.001
Smoking at the present						
	No	19,439.40	1,656	8.52	1.09 (1.05–1.12)	(Reference)
	Yes	4,782.69	410	8.57	1.09 (1.02–1.16)	0.500
Drinking at the present						
	No	19,417.00	1,602	8.25	1.09 (1.05–1.13)	(Reference)
	Yes	4,805.09	464	9.66	1.08 (1.02–1.15)	0.606
Exercising at the present
	No	16,774.29	1,389	8.28	1.10 (1.06–1.14)	(Reference)
	Yes	7,447.80	677	9.09	1.07 (1.02–1.12)	0.864
Self-reported diabetes						
	No	23,806.04	2,013	8.46	1.12 (1.09–1.16)	(Reference)
	Yes	416.05	35	8.41	1.20 (0.90–1.59)	0.337
Self-reported heart disease						
	No	22,814.56	1,958	8.58	1.12 (1.08–1.16)	(Reference)
	Yes	1,407.53	108	7.67	1.10 (0.99–1.23)	0.644
BMI index						
	18.5–23.9	13,282.30	1,143	8.61	1.09 (1.05–1.14)	(Reference)
	<18.5	8,242.74	647	7.85	1.03 (0.97–1.08)	0.974
	>23.9	2,697.05	276	10.23	1.07 (1.00–1.15)	0.761
Function disability
	Yes	3,341.052	137	4.10	1.04 (0.95–1.14)	(Reference)
	No	20,881.036	1,929	9.24	1.13 (1.09–1.17)	0.955
GDP per capita						
	< P_50_	12,129.68	1,065	8.78	1.30 (1.23–1.38)	(Reference)
	≥P_50_	12,092.41	1,001	8.28	1.05 (1.01–1.10)	0.999
The proportion of secondary industry						
	< P_50_	11,237.03	988	8.79	1.25 (1.18–1.32)	(Reference)
	≥P_50_	12,985.06	1,078	8.30	1.08 (1.04–1.13)	0.999

*The bold values mean statistic significance such as P = 0.001*.

## Discussion

We conducted a 10-year prospective cohort study to examine the association between PM_2.5_ exposure and the risk of hypertension incidence among Chinese elderly. Our results indicated that long-term exposure to PM_2.5_ was significantly associated with hypertension incidence among elderly individuals aged 65–116 years. We found that per 1 and 10 μg/m^3^ increase in PM_2.5_, the HR of hypertension incidence increased by 1 and 12% among the elderly, respectively. Similarly, there were strong positive links between Q2, Q3, and Q4 with PM_2.5_ exposure and hypertension incidence. Moreover, we observed that individuals with certain characteristics were more likely to be affected by PM_2.5_ exposure based on subgroup analysis.

We observed that the average exposure from 1-year before the event had the strongest effect on the incidence of hypertension, which was shorter than previous studies on the association between PM_2.5_ exposure and health outcomes have reported. For instance, some studies reported that a 3-year average PM_2.5_ exposure was most closely related to mortality (32, 37). Similarly, other studies have verified that an exposure period of <2 years might greatly influence cardiovascular health outcomes (38, 39). Thus, our results indicated that the impact of PM_2.5_ exposure was greater than we expected. The findings of our study suggest that interventions to improve air quality may reduce the incidence of hypertension within a short period.

According to the shape of the dose-response relationship between PM_2.5_ and hypertension incidence, we found that there was no safe threshold for PM_2.5_ exposure (Figure 3). Some studies have detected a threshold for PM_2.5_ exposure and negative health outcomes (22, 40), for example, a J-shaped association existed between PM_2.5_ exposure with a threshold concentration of 33 mg/m^3^, and function disability among the elderly. Other studies report that there is no safe threshold for PM_2.5_ exposure when considering asthma mortality and all-cause mortality risk (32, 41). Currently, the evidence for safe PM_2.5_ exposure with respect to hypertension incidence is limited. According to our findings, even with exposure to low levels of PM_2.5_, the risk of hypertension should not be overlooked. From a public policy perspective, reducing each one unit concentration of PM_2.5_ may be effective for protecting the elderly. Therefore, stricter national policy actions are recommended to improve air quality.

The adverse effects of PM_2.5_ exposure and increased risk for hypertension found in our study are in line with previous studies conducted among middle-aged and elderly people under 80 years of age (19, 21). For example, Wu et al. used a database including 20,927 middle-aged and older participants and reported that the increase in hypertension incidence risk per unit PM_2.5_ exposure was about 4.8 and 6.3% higher among males and females, respectively (19). Lin et al. enrolled 59,456 adults aged 50 years and older from four cohorts in China and showed that each 10 μg/m^3^ increase in PM_2.5_ concentration increased the HR of hypertension incidence by 14 % (17). In addition, the current study showed that the HRs for Q2, Q3, and Q4 for PM_2.5_ exposure were relatively higher than those in other published studies (12). One explanation for this finding was that these participants in our study were older than other studies (mean age was 87.7 years in our paper). Expert proposes that the ability to adapt to air pollution exposure may be reduced due to aging and health status (22). However, one study that investigated elderly individuals residing in Taiwan found no significant association between PM_2.5_ exposure and hypertension (16). Discrepant results were also observed in a Black Women's Health Study that found PM_2.5_ exposure was not significantly associated with measured hypertension among middle-aged and elderly women (9). In the present study, the mean of PM_2.5_ exposure concentration (55.3 μg/m^3^) was relatively higher than PM_2.5_ concentration from other studies conducted in the USA (8.6 μg/m^3^) (7), Seoul (38.9 μg/m^3^) (42), Europe (range: 5.0–8.9 μg/m^3^) (43), and a cross-sectional study from India (33.0 μg/m^3^) (11). Our finding was supported by a prior paper reported that the risk of PM_2.5_ exposure was declining in most western developed countries; in contrast, the risk of PM_2.5_ exposure was prominent in developing countries (44). Since 2013, the Chinese government has carried out a pilot project to monitor PM_2.5_ concentration in 33 major cities and implemented an air quality standard for PM_2.5_ concentration in 2016 (45). In 2019, the PM_2.5_ concentration in 22 of 31 major cities in mainland China exceeded the annual limitation concentration for China (35.0 μg/m^3^) and 31 of them exceeded the WHO recommendation (10.0 μg/m^3^) (46). Overall, the regional development patterns and air pollutant concentrations varies across countries and regions, which may explain some of the heterogeneity of PM_2.5_ exposure; the inconsistency between our results and those of other studies may also be caused by differences in time spent outdoors, population structures, and accessibility to health care (32).

To date, relevant studies have been proposed to explain the impacts of PM_2.5_ exposure on blood pressure through several mechanisms. One study posits that PM_2.5_ is taken into the human body by direct translocation through the olfactory bulb, leading to inflammatory responses and oxidative stress (47). Another proposes that inhaled particles destroy the balance in the autonomic nervous system, inducing a sympathetic response, followed by arterial vasoconstriction (48). Finally, it may be that long-term exposure to PM_2.5_ affect the systemic hemodynamics of the body due to endothelial injury or dysfunction, which is also considered a risk factor for hypertension (10). In summary, the beneficial efforts to reduce PM_2.5_ emission on hypertension among the elderly would be important.

Our findings implied that the association between each 10-μg/m^3^ increment in PM_2.5_ exposure and hypertension was modified by different characteristics of the elderly. We found that HR seemed more apparent in individuals without pensions. One study concluded that the health impact of PM_2.5_ on individuals with lower socioeconomic status was slightly greater than that of others (49). In our study, most of the participants were uneducated and had no pension, so they were probably more likely to spend their time engaging in outdoor manual labor that more easily suffered from PM_2.5_ exposure. Additionally, the subgroup analysis showed that respondents who lived in rural areas and central/western China were more likely to have hypertension. According to the distribution of PM_2.5_ concentration in mainland China in this study (Figure 2), we found most of the central or western areas such as Szechwan, Hunan, and Hubei provinces, and the Beijing-Tianjin-Hebei region had higher air pollutant levels. It is important to note that these areas are traditional industrial districts with a high population density. In rural areas, the PM_2.5_ concentration was higher than in urban areas probably due to residential energy materials such as coal and biomass fuels. Accordingly, several approaches, such as clean energy use in rural areas and upgrading of traditional industries in traditional industrial districts, would improve air quality and decrease the risk of hypertension among the elderly.

Some limitations of this study should be acknowledged. First, according to the diagnostic guide, antihypertensive medication was one of the criteria for hypertension diagnosis, but the information was not available in the CLHLS database. In our study, we used blood pressure values and self-reported history of hypertension diagnosis to identify hypertension, which might be affected by recall bias. However, it could be confirmed that individuals diagnosed with hypertension by a physician should be prescribed antihypertensive medication (16). The address of each participant was deleted from the CLHLS database and we could not collect the precise addresses of participants from this publicly open database. However, our study estimated PM_2.5_ exposure in residential counties or district addresses using satellite observations and an atmospheric chemistry database that effectively employed relatively coarse spatial resolution to estimate large-area variations in air pollution exposures (11, 50). Besides, lots of factors such as economic status, disease comorbidities, and life styles, which were dynamic and not easy to assess. Lastly, because the concentrations of other air pollutants such as SO_X_, NO_X_, and O_3_ were not unavailable in the open database, we could not confirm the effects of PM_2.5_ exposure and hypertension incidence under the influence of these air pollutants. A more comprehensive evaluation should be conducted to verify the influence of various air pollutants on hypertension in future studies.

Despite these limitations, this study further added to the present evidence of the effects of PM_2.5_ exposure on hypertension among the elderly. In particular, the study provided quantitative evidence relying on a 10-year period using a large population-based cohort that included a nationwide representative sample of a specific age group (≥80 years) in a developing country. The results can be used to inform public policy to address the urgent need for more rigorous environment-related policies for air pollution abatement and make efforts to protect vulnerable elderly away from such harmful exposure.

## Conclusion

This study highlighted the effects of long-term exposure to PM_2.5_, which was associated with hypertension incidence among the elderly, and indicated that there was no safe threshold for the association between PM_2.5_ and risk of hypertension. Furthermore, this relationship was greater in the elderly who were without pension, living in rural areas, and residing in central/western China. Reduction approaches and policies for PM_2.5_ should be developed to reduce the incidence of hypertension and improve the quality of life of the elderly.

## Data Availability Statement

The CLHLS questionnaires are available at https://sites.duke.edu/centerforaging/programs/chinese-longitudinal-healthy-longevity-survey-clhls/. The full datasets used in this analysis are available from the corresponding author upon reasonable request.

## Ethics Statement

The studies involving human participants were reviewed and approved by the Research Ethics Committees of Duke University and Peking University approved the protocol for each wave of the CLHLS, which is the data source of this study. The survey respondents gave informed consent before participating. The patients/participants provided their written informed consent to participate in this study.

## Author Contributions

ZW and ZF: data cleaning. ZW, ZF, CW, and LL: conceptualization and visualization. ZW, CW, ZF, and WW: data curation, writing, original draft preparation, methodology, software, and reviewing. ZW, ZF, CW, LL, and WW: supervision. All authors contributed to the article and approved the submitted version.

## Funding

The research was financially supported by National Natural Science Foundation of China (Grant Nos. U1911204 and 51861125203), National Key R&D Program of China (2017YFC0405900), and the Project for Creative Research from Guangdong Water Resources Department (Grant Nos. 2018, 2020).

## Author Disclaimer

The views expressed in this article belong to the authors and are not to the official position of any other institution or funder.

## Conflict of Interest

The authors declare that the research was conducted in the absence of any commercial or financial relationships that could be construed as a potential conflict of interest.

## Publisher's Note

All claims expressed in this article are solely those of the authors and do not necessarily represent those of their affiliated organizations, or those of the publisher, the editors and the reviewers. Any product that may be evaluated in this article, or claim that may be made by its manufacturer, is not guaranteed or endorsed by the publisher.
