# Supplementary material for: The Association Between Long-Term Exposure to Particulate Matter and Incidence of Hypertension Among Chinese Elderly: A Retrospective Cohort Study
Source: Front Cardiovasc Med. 2022 Jan 11;8:784800. doi: 10.3389/fcvm.2021.784800 (PMC8788195; doi:10.3389/fcvm.2021.784800)
Supplement: Supplementary file 1 [file Table_1.docx]

**Supplementary material**

**Table S1 Window analyses for the association between average baseline, 1-, 2-, 3-, 4-, 5-, 6-, 7- year before events or last interview exposure of PM_2.5_ (each 10 µg/m^3^) and hypertension incidence among the elderly**

| PM 2.5 exposure | Model 1 ^a^ |
| --- | --- |
| Baseline average | **1.05(1.01-1.08)** |
| 1-year average | **1.12(1.09-1.16)** |
| 2-year average | **1.10 (1.06-1.14)** |
| 3-year average | **1.08 (1.04-1.12)** |
| 4-year average | **1.09 (1.05-1.13)** |
| 5-year average | **1.07 (1.03-1.10)** |
| 6-year average | **1.04 (1.00-1.08)** |
| 7-year average | 0.99 (0.95-1.01) |

a Random-effects Cox proportional hazards model for the sampling sites adjusted for age, gender, education attainment, pension,living arrangement, marital status, regions, residence, smoking at the present, drinking at the present, exercising at the present, BMI index, self-reported diabetes, function disability, GDP per capita, the proportion of secondary industry and heart disease.

**Table S2 The correlation between annual average exposure of PM_2.5_**

|  | **Baseline exposure** | **Average 1-year exposure** | **Average 2-year exposure** | **Average 3-year exposure** | **Average 4-year exposure** | **Average 5-year exposure** | **Average 6-year exposure** | **Average 7-year** **exposure** |
| --- | --- | --- | --- | --- | --- | --- | --- | --- |
| **Baseline exposure** | 1 |  |  |  |  |  |  |  |
| **Average 1-year exposure** | 0.915****** | 1 |  |  |  |  |  |  |
| **Average 2-year exposure** | 0.939****** | 0.983****** | 1 |  |  |  |  |  |
| **Average 3-year exposure** | 0.948****** | 0.973****** | 0.993****** | 1 |  |  |  |  |
| **Average 4-year exposure** | 0.965****** | 0.984****** | 0.993****** | 0.992****** | 1 |  |  |  |
| **Average 5-year exposure** | 0.955****** | 0.958****** | 0.978****** | 0.991****** | 0.984****** | 1 |  |  |
| **Average 6-year exposure** | 0.951****** | 0.945****** | 0.967****** | 0.982****** | 0.975****** | 0.996****** | 1 |  |
| **Average 7-year exposure** | 0.957****** | 0.949****** | 0.969****** | 0.982****** | 0.978****** | 0.995****** | 0.997****** | 1 |

**Note: * P<0.05; ** P<0.01**

**Table S3 Sensitivity analysis for the association between PM_2.5_ (each 10 µg/m3) and hypertension incidence among the elderly**

| **Sensitivity analysis** | **Model 1 ^a^** |
| --- | --- |
| Excluding participants dead during follow-up and lost follow-up (i.e. complete follow-up experience) | **1.12(1.09-1.16)** |
| Excluding participants dead during follow-up | **1.12(1.09-1.16)** |
| Excluding participants with address change | **1.12(1.09-1.16)** |

a Random-effects Cox proportional hazards model for the sampling sites adjusted for age, gender, education attainment, pension,living arrangement, marital status, regions, residence, smoking at the present, drinking at the present, exercising at the present, BMI index, self-reported diabetes, function disability, GDP per capita, the proportion of secondary industry and heart disease.
